# Neural stem cell therapy for stroke: A multimechanistic approach to restoring neurological function

**DOI:** 10.1002/brb3.1214

**Published:** 2019-02-12

**Authors:** Emily W. Baker, Holly A. Kinder, Franklin D. West

**Affiliations:** ^1^ Regenerative Bioscience Center University of Georgia Athens Georgia; ^2^ Department of Animal and Dairy Science University of Georgia Athens Georgia

**Keywords:** cell replacement, Neural stem cell, neuroprotection, regenerative medicine, stroke

## Abstract

**Introduction:**

Neural stem cells (NSCs) have demonstrated multimodal therapeutic function for stroke, which is the leading cause of long‐term disability and the second leading cause of death worldwide. In preclinical stroke models, NSCs have been shown to modulate inflammation, foster neuroplasticity and neural reorganization, promote angiogenesis, and act as a cellular replacement by differentiating into mature neural cell types. However, there are several key technical questions to address before NSC therapy can be applied to the clinical setting on a large scale.

**Purpose of Review:**

In this review, we will discuss the various sources of NSCs, their therapeutic modes of action to enhance stroke recovery, and considerations for the clinical translation of NSC therapies. Understanding the key factors involved in NSC‐mediated tissue recovery and addressing the current translational barriers may lead to clinical success of NSC therapy and a first‐in‐class restorative therapy for stroke patients.

## 1 INTRODUCTION

Although stroke is the leading cause of long‐term disability and the second leading cause of death worldwide, there are only two Food and Drug Administration (FDA)‐approved therapies—tissue plasminogen activator and thrombectomy (Albers et al., 2018; Mozaffarian et al., 2015; Nogueira et al., 2018; Sharma et al., 2010). However, these therapies are significantly limited as they can only be utilized in acute patients resulting in a relatively small number of individuals being treated. Most therapies recently tested in clinical trials have focused on mitigating secondary injury mechanisms such as excitotoxicity (Clark, Wechsler, Sabounjian, & Schwiderski, 2001; Diener et al., 2000, 2008; Mousavi, Saadatnia, Khorvash, Hoseini, & Sariaslani, 2011), immune and inflammatory responses (Enlimomab Acute Stroke Trial & I., 2001), or apoptosis (Franke et al., 1996), all of which have failed. Neural stem cells (NSCs) have garnered significant interest as a multimodel therapeutic capable of producing neuroprotective and regenerative growth factors, while also potentially serving as cell replacement for lost and damaged neural cell types (Andres et al., 2011; Baker et al., 2017; Chang et al., 2013; Eckert et al., 2015; Tornero et al., 2013; Watanabe et al., 2016; Zhang et al., 2011). Another potentially attractive advantage of NSC therapy over conventional drug therapies is NSCs can continually respond to environmental cues and secrete appropriate quantities and type of signaling factors, therefore providing a tailored response to individual stroke injuries. Due to the significant potential of NSCs, these cells have progressed from testing in preclinical models to clinical trials for stroke with promising results (Table 1; Andres et al., 2011; Kalladka et al., 2016; Watanabe et al., 2016; Zhang et al., 2011, 2013). NSCs are multipotent and specifically differentiate into neural cell types (e.g., neurons, astrocytes and oligodendrocytes) and thus likely hold the greatest potential for cell replacement therapy after stroke. While significant progress has been made to understand NSC‐mediated tissue recovery after stroke, key questions remain that must be resolved before NSC therapy can be utilized in the clinic at a large scale. In this review, we will discuss the sources of NSCs currently being studied, their mode of action in the context of stroke treatment, and clinical considerations to move NSC therapies from human trials to a standard of care for stroke patients.

**Table 1:** Preclinical rodent ischemic stroke models testing human neural stem cell therapy

NSC type	Transplantation time point post‐stroke	Route of administration	Cell dose	Modes of action identified	Reference
Fetal‐derived	1 week	IP	3 × 100,000	Cell replacement Synaptic reorganization	Andres et al. (2011)
Fetal‐derived	6 hr	IV	1 × 3,000,000	Immunomodulation	Watanabe et al. (2016)
Fetal‐derived	1 day	IP	1 × 100,000	Immunomodulation	Huang et al. (2014)
Fetal‐derived	1–2 weeks	IP	2 × 150,000	Cell replacement	Darsalia et al. (2007)
Fetal‐derived	1 day	IV	1 × 4,000,000	Cell replacement Neuroprotection Angiogenesis	Song et al. (2015)
Fetal‐derived	1 week	IP	3 × 100,000	Cell replacement Immunomodulation	Kelly et al. (2004)
Fetal‐derived	4 weeks	IP	2 × 225,000; 1 × 4.5 × 10^3^, 4.5 × 10^4^, or 4.5 × 10^5^ a	Neurogenesis Angiogenesis	Hassani et al. (2012), Hicks et al. (2013) and Stroemer et al. (2009)
Fetal‐derived	3 weeks, 2 days^a^	IP	2 × 100,000	Cell replacement Neurogenesis Immunomodulation	Mine et al. (2013)
Fetal‐derived	1 day	ICV	1 × 120,000	Cell replacement Neuroprotection Neurogenesis Angiogenesis	Ryu et al. (2016)
hESC‐derived	1 day	IP	1 × 50,000	Neurogenesis Angiogenesis	Zhang et al. (2011)
hESC‐derived	1 week	IP	1 × 200,000	Cell replacement Immunomodulation	Chang et al. (2013)
hESC‐derived	2 weeks	IP	1 × 120,000	Cell replacement Neurogenesis	Jin et al. (2011)
iPSC‐derived	Immediately after stroke reperfusion	IP	1 × 1,000,000	Cell replacement	Yuan et al. (2013)
iPSC‐derived	1 week	IP	Mouse: 1 × 100,000 Rat: 2 × 200,000 or 2 × 150,000^a^	Cell replacement Angiogenesis	Oki et al. (2012)
iPSC‐derived	1 week	IP	1 × 100,000	Cell replacement Neuroprotection	Polentes et al. (2012)
iPSC‐derived	2 days	IP	2 × 150,000	Cell replacement	Tornero et al. (2013)
iPSC‐derived	1 week	IP	1 × 200,000	Cell replacement Immunomodulation Neurogenesis	Zhang et al. (2013)
iPSC‐derived	1 day	IP	1 × 100,000	Immunomodulation	Eckert et al. (2015)

Notes

hESC: human embryonic stem cell; ICV: intracerebroventricular; IP: intraparenchymal; iPSC: induced pluripotent stem cell; IV: intravenous; NSC: neural stem cell.

a two separate experiments were performed. Cell dosing nomenclature is as follows: [number of injection sites] × [number of NSCs per injection]. For each experiment, all cell injections were performed on the same day.

## 2 SOURCES OF NEURAL STEM CELLS

**Figure 1:**
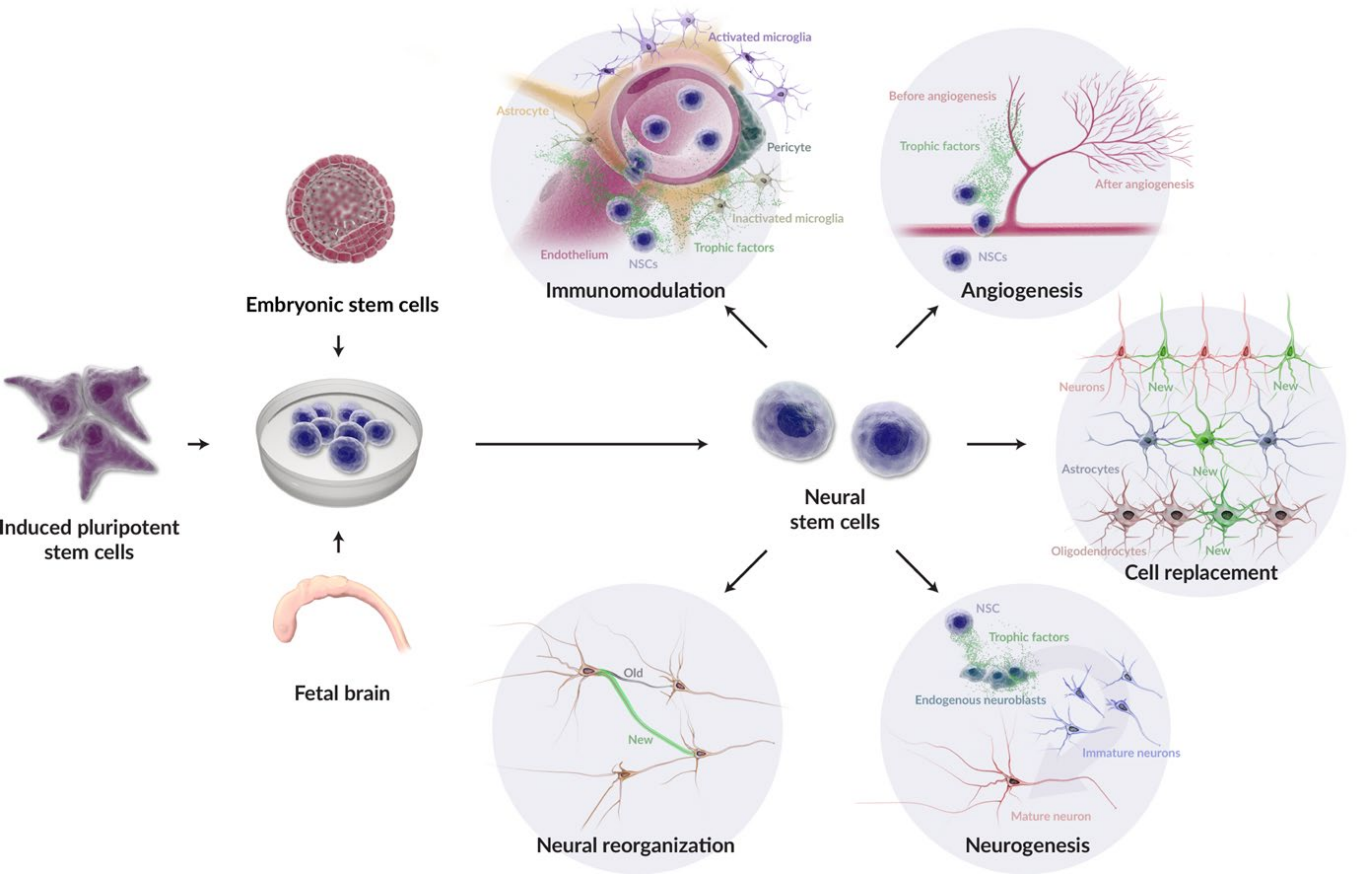
Multifunctional therapeutic action of transplanted neural stem cells. Transplanted NSCs derived from ESCs, iPSCs, or fetal brain have demonstrated multimodal therapeutic function after intravenous, intraparenchymal, or intracerebroventricular administration (Kokaia et al., 2012; Lau et al., 2015). NSCs demonstrate immunomodulatory function through the expression of cytokines and chemokines in response pro‐inflammatory signaling from activated microglia and infiltrating circulatory immune cells (Huang et al., 2014; Watanabe et al., 2016). NSCs also promote angiogenesis and stimulate neural repair mechanisms including synaptic reorganization and neurogenesis (Andres et al., 2011; Zhang et al., 2011). Transplanted NSCs can act as a cell replacement therapy by differentiating to mature neural cell types (neurons, astrocytes, and oligodendrocytes) and integrating into the host brain tissue (Baker et al., 2017; Kelly et al., 2004; Oki et al., 2012; Tornero et al., 2013). The prepotency of one mechanism to promote tissue repair over another is not well known. Regardless of therapeutic mechanism, the final outcome after NSC transplantation is improved tissue and functional recovery (Kokaia et al., 2012)

During the 1990s, novel protocols were developed to generate immortalized human neural cell lines capable of differentiating into mature neurons on a scale large enough to be therapeutically relevant (Carpenter et al., 1999; Storch et al., 2001; Svendsen et al., 1998; Villa, Snyder, Vescovi, & Martínez‐Serrano, A., 2000). Since then, multiple types of NSC lines that generate mature neural cell types have been developed and characterized including NSCs derived from fetal tissues, embryonic stem cells (ESCs), and induced pluripotent stem cells (iPSCs), all of which have been shown to enhance recovery after stroke and comparable neurological disorders (Figure 1).

### 2.1 Fetal‐derived neural stem cells

Fetal‐derived NSC lines (fetal‐NSC) were one of the first cell sources developed that had significant potential as a stroke cell therapy. Fetal‐NSCs were generated by dissociating human fetal cortex, mesencephalon, or spinal cord tissues between 7 and 21 days postconception (Pollock et al., 2006; Svendsen et al., 1998). These cells proved to be capable of long‐term expansion when cultured in mitogens such as epidermal growth factor (EGF) and fibroblast growth factor‐2 (FGF‐2), even without genetic modification, while maintaining their neurogenic and gliogenic multipotent differentiation potential. Fetal‐NSCs have shown therapeutic potential for a number of neurological diseases such as stroke (Kalladka et al., 2016; Pollock et al., 2006), traumatic brain injury (Shin et al., 2015; Skardelly et al., 2011), spinal cord injury (Cheng et al., 2012; Shin et al., 2015), and amyotrophic lateral sclerosis (Glass et al., 2012; Xu et al., 2006). However, previous studies have shown that fetal‐NSCs undergo senescence earlier than other NSC lines, which would make large‐scale manufacturing more difficult (Wright, Prowse, Wallace, Linskens, & Svendsen, 2006). To address this, some fetal‐NSC lines have undergone genetic modification leading to immortalization and enhanced expandability. A prominent example of this is the CTX0E03 cell line, which is derived from fetal cortical tissue and transduced with a *c*‐mycER^TAM^ construct (Pollock et al., 2006). Studies have demonstrated that CTX0E03 has multimodal therapeutic function in preclinical animal models including angiogenic, neurogenic, and immunomodulation effects leading to improvements in functional recovery (Hassani et al., 2012; Hicks et al., 2013; Stroemer et al., 2009). CTX0E03 was recently evaluated in phase I clinical trials for stroke and demonstrated no cell‐related adverse events (Hassani et al., 2012; Hicks et al., 2013; Kalladka et al., 2016; Stroemer et al., 2009). However, these cells have shown limited long‐term engraftment suggesting that the effect of these cells is mostly due to paracrine signaling and not cell replacement (Hicks et al., 2013). Limited cell replacement is a common challenge in NSC therapy regardless of cell source or method of delivery (e.g., intraparenchymal, intravenous). This suggests that an increased level of efficacy could be achieved with NSC therapy if cells could be maintained long term and successfully integrate into damaged tissues. The cause of limited NSC engraftment is still unclear, but is likely a combination of a number of factors including the hostile stroke environment (e.g., high levels of reactive oxygen species and inflammatory cytokines) and the lack of appropriately orchestrated differentiation and integration signaling.

### 2.2 Embryonic stem cell‐derived neural stem cells

After the establishment of cultured embryonic stem cell (ESC) lines (Thomson, 1998), several groups have successfully developed ESC‐derived long‐term expandable NSCs (ESC‐NSCs) from multiple species including human (Elkabetz et al., 2008), mouse (Lee, Lumelsky, Studer, Auerbach, & McKay, 2000), and primate (Ikeda et al., 2005). The therapeutic use of ESC‐NSCs could be advantageous over NSCs of fetal origin due to the theoretically limitless source of immortal ESCs in which to scale‐up cell manufacturing to meet the clinical demand (Manley, Azevedo‐Pereira, Bliss, & Steinberg, 2015). To date, there have been no clinical trials testing ESC‐NSCs for stroke. However, a phase I clinical trial testing ESC‐NSCs was recently completed for spinal cord injury (clinicaltrials.gov ID: NCT01217008), overcoming ethical and regulatory challenges and paving the way for future ESC‐NSC stroke clinical trials (Daadi & Steinberg, 2009).

### 2.3 Induced pluripotent stem cell‐derived neural stem cells

More recently, the research of Yamanaka and others have shown that adult somatic cells can be reprogrammed to a pluripotent state through the overexpression of transcription factors, and these reprogrammed cells have similar plasticity and neural differentiation potency as ESCs (Denham & Dottori, 2011; Takahashi et al., 2007; Warren et al., 2010). These cells have been deemed induced pluripotent stem cells (iPSCs), and their NSC progeny (iPSC‐NSCs). iPSC‐NSCs possess unprecedented therapeutic potential for neurological disease as they can be generated from the patient's own somatic cells, avoiding the risk of immune rejection associated with allogeneic transplants (Araki et al., 2013; Guha, Morgan, Mostoslavsky, Rodrigues, & Boyd, 2013). Since the in vitro characterization of iPSC‐NSCs, several studies have documented improved tissue and functional recovery after transplantation into preclinical rodent and pig stroke models (Oki et al., 2012; Polentes et al., 2012; Table 1). Oki et al. demonstrated enhanced neural plasticity likely mediated by increased vascular endothelial growth factor (VEGF) expression in the brain of iPSC‐NSC‐grafted rodents, which was associated with improved fine forelimb movement indicated by the staircase test (Oki et al., 2012). Furthermore, Polentes et al. (2012) showed that transplanted iPSC‐NSCs differentiated into site‐specific neuronal cells which functionally incorporated into host circuitry and prevented stroke‐associated neurological deficits in rats. More recently, Baker et al. (2017) documented mitigated immune response after iPSC‐NSC transplant into the stroke‐damaged pig brain which was associated with improved white matter integrity, neurometabolite abundance, and cerebral blood perfusion. iPSC‐NSC‐treated stroke pigs ultimately demonstrated faster recovery of functional deficits relative to nontreated control stroke pigs (Lau, Platt, Grace, Baker, & West, 2018). Although further progress is needed for clinical application of iPSC technology for stroke, with shortened reprogramming duration being critical, these preclinical studies show that iPSC‐NSCs are a promising future treatment option for stroke (Lau, Platt, Stice, & West, 2015).

## 3 NEURAL STEM CELL MODES OF ACTION

NSCs have demonstrated multimodal therapeutic function after transplantation into preclinical animal models of stroke (documented NSC modes of action in preclinical animal models are summarized in Table 1). Depending on the treatment protocol, NSCs are able to protect at‐risk neural cells, promote endogenous NSC proliferation and migration, foster synaptic remodeling, stimulate new vessel formation, and/or integrate into host neural circuits, which have been associated with improvements in cognitive and sensorimotor function (Mine et al., 2013; Zhang et al., 2013; Figure 1). However, further studies are needed to determine which tissue recovery mechanism is most effective in restoring neurological function.

### 3.1 Immunomodulation

Stroke injury is propagated by a strong immune and inflammatory response through the activation of microglia, the resident immune cells of the brain, which produce pro‐inflammatory cytokines such as interleukin (IL)‐1β, IL‐6, and tumor necrosis factor‐alpha (TNF‐α) in response to damage‐associated molecular patterns (DAMPS) within minutes after onset of ischemia (Hossmann, 2006; Jin, Yang, & Li, 2010; Xiong, Liu, & Yang, 2016). Furthermore, the release of cytokines by activated microglia upregulates the expression of chemokines such as monocyte chemoattractant protein‐1 (MCP‐1) and chemokine ligand 1 (CXCL1) on endothelial cells, which promotes the infiltration of peripheral monocytes/macrophages across the blood–brain barrier (BBB) which exacerbates inflammatory injury (Remus, Sayeed, Won, Lyle, & Stein, 2015). The modulation of this inflammatory cascade may be the most widely characterized NSC mode of action in preclinical animal models of stroke. The immunomodulatory mechanisms of transplanted NSCs are likely carried out through what is known as the “bystander effect,” in which NSCs release neurotrophic factors such as glial‐derived neurotrophic factor (GDNF), nerve growth factor (NGF), and brain‐derived neurotrophic factor (BDNF), which have been shown to inhibit mechanisms induced by inflammatory actions (Lladó, Haenggeli, Maragakis, Snyder, & Rothstein, 2004; Lu, Jones, Snyder, & Tuszynski, 2003; Ourednik, Ourednik, Lynch, Schachner, & Snyder, 2002). Multiple studies in preclinical stroke models have documented decreased immune cell activation after intraparenchymal transplantation of fetal and iPSC‐NSCs (Eckert et al., 2015; Kelly et al., 2004; Mine et al., 2013; Zhang et al., 2013). Ultimately, reduced inflammation mediated by NSC transplantation is neuroprotective because the secondary injury cascade is curtailed; thus, reports of reduced inflammation in the stroked brain are often correlated with reductions in final infarct volume and functional recovery (Bacigaluppi et al., 2009; Huang, Wong, Snyder, Hamblin, & Lee, 2014; Song et al., 2015).

More studies are needed to assess whether one NSC type possesses heightened immunomodulatory properties over another. Experiments performed by the Lee group showed decreases in the expression of pro‐inflammatory cytokines TNF‐α, IL‐6, IL‐1β, cell adhesion molecules ICAM‐1 and VCAM‐1, and chemokines MCP‐1 and MIP‐1α after intraparenchymal transplantation of fetal‐ and iPSC‐NSCs in a rodent stroke model (Eckert et al., 2015; Huang et al., 2014). This decrease in pro‐inflammatory cytokines and chemokines was attributed, at least partially, to NSC‐mediated increases in BDNF expression in the ipsilateral hemisphere. These data indicate that neurotrophic factor signaling by transplanted NSCs of both iPSC and fetal origin diminishes upstream inflammatory activity in the early stages of ischemic injury by reducing pro‐inflammatory signaling by activated microglia, which in turn inhibits subsequent peripheral immune cell extravasation. Indeed, the documented changes in gene expression correlated to reduced expression of the microglia marker Iba1 and reduced BBB leakage. However, fetal‐NSCs were more effective at reducing final infarct volume compared to iPSC‐NSCs, which may be due to differences in the neurotrophic signaling capacity of the two cell types.

Although NSC immunomodulatory behavior in the stroked brain is often documented after an intraparenchymal transplantation approach, there is evidence that intravenous delivery of NSCs after stroke also reduces the expression of pro‐inflammatory mediators, immune cell activation, and apoptosis in brain tissue (Bacigaluppi et al., 2009; Song et al., 2015; Watanabe et al., 2016). Whether it is necessary for these cells to accumulate in the brain parenchyma after intravenous delivery in order to carry out their anti‐inflammatory effects is largely unknown. However, a study by Song et al. (2015) demonstrated that intravenously delivered, magnetically targeted fetal‐NSCs accumulated in the stroked brain tissue and correlated to reduced infarct size compared to nontargeted NSCs. Taken together, these data indicate that targeting systemically infused NSCs to the brain parenchyma augments their immunomodulatory capacity after stroke. Therefore, direct intraparenchymal transplantation may be advantageous over intravenous delivery to enhance NSC‐mediated tissue repair.

### 3.2 Neuroplasticity and Reorganization

#### 3.2.1 Neurogenesis

For many years, it was widely accepted that the brain possessed no regenerative potential and lost neurons could not be replaced after injury or disease. However, more recently, it has been demonstrated that endogenous NSCs are present in the subventricular zone (SVZ; Corotto, Henegar, & Maruniak, 1993; Kirschenbaum et al., 1994) and the dentate gyrus (DG; Eriksson et al., 1998; Kuhn, Dickinson‐Anson, & Gage, 1996) of the adult mammalian brain, and neurogenesis in these regions is increased after stroke albeit at a level lower than what is necessary to restore tissue function (Arvidsson, Collin, Kirik, Kokaia, & Lindvall, 2002). Indeed, despite evidence of functional integration of these nascent neurons (Hou et al., 2008; Yamashita et al., 2006), about 80% of the new neuroblasts and neurons die within the first 2 weeks after formation (Arvidsson et al., 2002). The potential to enhance endogenous neurogenesis mechanisms to replace lost neuronal cells has been an exciting therapeutic target for stroke. Previous studies have shown that stroke‐induced neurogenic behavior is augmented after NSC transplantation in preclinical stroke models by increasing the proliferation of endogenous NSCs at the SVZ and DG as well as promoting the migration of endogenous neuroblasts to the damaged brain region which differentiate to mature neurons (Hassani et al., 2012; Jin et al., 2011; Mine et al., 2013; Ryu, Lee, Kim, & Yoon, 2016; Stroemer et al., 2009; Zhang et al., 2011, 2013). A study by Mine et al. (2013) illustrated that intraparenchymal transplantation of fetal‐NSCs 2 days after stroke increased both the number of proliferating cells in the SVZ and the number of endogenous neuroblasts in the injured striatum up to 14 weeks after stroke. Furthermore, these newly produced endogenous neuronal cells colabeled with the mature neuron marker Fox‐3, which indicates that NSC transplantation augments several steps of striatal neurogenesis after stroke. Interestingly, in this study the neurogenic activity seemed to be enhanced when NSCs were transplanted 2 days after stroke compared to 3 weeks after stroke. This suggests that timing of transplantation after injury plays an important role in the enhancement of endogenous neurogenesis by transplanted NSCs. However, there are discrepancies among preclinical studies with respect to duration of enhanced neurogenesis, which may be due to differences in the stroke models, transplantation location, and neurogenic potency of the cell line tested. The mechanism by which transplanted NSCs augment endogenous neurogenic behavior after stroke is not well known, but is likely mediated by the secretion of neurotrophic and regenerative growth factors that suppress immune and inflammatory responses while promoting tissue repair (Kokaia, Martino, Schwartz, & Lindvall, 2012; Lu et al., 2003; Martino & Pluchino, 2006).

#### 3.2.2 Neural reorganization

Patients show varying levels of spontaneous recovery in limb function, language, and other cognitive measures within the first month after stroke onset (Benowitz & Carmichael, 2010). This phenomenon is largely attributed to the rewiring of neuronal circuitry in which motor and sensory circuit activity is increased in other brain regions remote from the infarcted area (Cramer, 2008). This reorganization is modulated by structural changes in axons, dendrites, and synapses as well as increased activation of endogenous NSCs. More recently, preclinical animal models have documented that synaptic reorganization is augmented by NSC transplantation after stroke. In a study by Patkar, Tate, Modo, Plevin, and Carswell (2012), intraparenchymal transplantation of a murine NSC line led to increased expression of the synaptogenesis marker synaptophysin and the axonal growth cone protein GAP‐43 in the stroked brain which was partially expressed by the transplanted cells themselves. Furthermore, other studies have demonstrated that intraparenchymal transplantation of fetal‐ and ESC‐NSCs augments dendritic plasticity (including increased dendritic length and branching) in the cortex and axonal sprouting in the cortex, striatum, thalamic nuclei, and corpus callosum after stroke which was associated with recovery in motor function (Andres et al., 2011; Daadi et al., 2010). The observed synaptic reorganization events were associated with transplanted NSC‐induced increases in the expression of genes involved in neurite outgrowth including thrombospondins (TSPs) 1 and 2, VEGF, glial‐derived neurotrophic factor (GDNF), neurturin, and insulin growth factor‐1 (IGF‐1). Furthermore, immunodepletion studies of NSCs in vitro demonstrated that a subset of these factors, TSP1, TSP2, and VEGF, were specifically secreted by NSCs and were responsible for NSC‐induced effects on dendritic and axonal plasticity (Andres et al., 2011). Other studies in preclinical stroke models have demonstrated that transplanted NSCs promote oligodendrocyte proliferation and myelination of new neuronal circuits (Daadi et al., 2010; Manley et al., 2015; Stroemer et al., 2009; Zhang et al., 2013). These results show that NSC transplantation augments key reorganization of axons, dendrites, and synapses across multiple brain regions that lead to improved recovery.

#### 3.2.3 Angiogenesis

Surrounding the ischemic core, a region of unsalvageable damaged tissue, is an area of mild‐to‐moderately hypoperfused tissue known as the penumbra (Arai, Jin, Navaratna, & Lo, 2009; Arai et al., 2011). The penumbra is impacted by the ischemic event but still viable due to collateral blood flow and thus potentially salvageable. Increasing microvascularization in the penumbra region is a therapeutic target that could increase survival of the tissue surrounding the infarct and improve functional outcome after stroke (Morris et al., 2003). After stroke, vascular regrowth is stimulated in part by the exchange of neurotrophic and regenerative growth factors between local endothelial cells and endogenous NSCs which have migrated to the site of ischemic insult. This cross talk between neural and vascular compartments has been shown to promote angiogenesis in preclinical stroke models and human stroke patients leading to improved functional outcome (Arai et al., 2011; Ding et al., 2008; Horie et al., 2011; Krupiński, Kałuza, Kumar, Kumar, & Wang, 1993; Krupinski, Kumar, Kumar, & Kaluza, 1996; Wang et al., 2012). Previous studies in rodents have documented that transplanted NSCs increase endothelial cell proliferation, microvessel density, and angiogenic receptor expression in the penumbra region of stroked brain tissue indicative of enhanced angiogenesis (Horie et al., 2011; Ryu et al., 2016; Song et al., 2015; Stroemer et al., 2009; Zhang et al., 2011). It is well characterized that NSC‐mediated angiogenic behavior is largely modulated via VEGF signaling, secreted either by NSCs themselves or enhanced expression by host tissue (Arai et al., 2009; Horie et al., 2011; Lee, Kim, Park, & Kim, 2007; Oki et al., 2012). These studies have shown strong correlations between increases in VEGF signaling to increased penumbra vascularization and functional recovery. In addition to VEGF, Hicks et al. (2013) demonstrated that fetal‐NSCs express the angiogenesis signaling factor Ang1 and lead to increased numbers of microvessels after transplantation in the stroked brain. The formation of robust nascent vessel networks in the penumbra require the creation of functional neurovascular units (NVU) that maintain cerebral blood perfusion, regulate homeostasis, and are selectively permeable (Arai et al., 2009). Horie et al. (2011) demonstrated that NSC treatment leads to enhanced NVU formation as indicated by increased expression of tight junction proteins (claudin, occludin, and ZO1) and dystroglycan, a protein involved in binding astrocytic endfeet to endothelial cells, and reduced BBB leakage post‐stroke in penumbral tissue. Together, these studies indicate that neovascularization is a major mechanism of NSC‐mediated tissue and functional recovery after stroke.

### 3.3 Cell Replacement

In addition to the many mechanisms in which NSC‐mediated trophic factor signaling protects and promotes tissue recovery after stroke, NSCs themselves can act as a cellular replacement. After transplantation into the stroke‐damaged brain, grafted NSCs have been shown to stop proliferating between 2–8 weeks post‐transplant and express the neuroblast marker doublecortin by 2 months post‐transplant, which eventually diminishes as cells terminally differentiate into neurons or glia (Darsalia, Kallur, & Kokaia, 2007; Jin et al., 2011; Oki et al., 2012). Many studies have documented terminal differentiation of NSCs into neurons in the stroke‐damaged brain that express neuron markers such as NeuN, HuD, MAP2, and βIII‐tubulin (Chang et al., 2013; Darsalia et al., 2007; Mine et al., 2013; Oki et al., 2012; Song et al., 2015; Tornero et al., 2013; Zhang et al., 2013). Grafted NSC‐derived neurons are capable of differentiating into a wide array of mature neuron subtypes expressing dopaminergic neuron (Chang et al., 2013; Polentes et al., 2012; Zhang et al., 2013), GABAergic interneuron (Darsalia et al., 2007; Oki et al., 2012; Polentes et al., 2012; Zhang et al., 2013), medium spiny striatal projection neuron (Chang et al., 2013; Oki et al., 2012; Polentes et al., 2012; Zhang et al., 2013), and cortical neuron markers (Darsalia et al., 2007; Oki et al., 2012; Tornero et al., 2013). Furthermore, some of these studies demonstrate that grafted NSC‐derived neurons are electrically active, project axons to appropriate target regions, and form synapses (Chang et al., 2013; Mine et al., 2013; Oki et al., 2012; Polentes et al., 2012; Tornero et al., 2013). Glial differentiation (astrocytes and oligodendrocytes) has also been documented with a similar time course to neuronal cells (Andres et al., 2011; Darsalia et al., 2007; Kelly et al., 2004; Oki et al., 2012; Song et al., 2015; Stroemer et al., 2009; Zhang et al., 2013). However, there is much debate whether transplanted NSCs truly integrate into the host brain and contribute directly to improving functional outcome in preclinical animal models. Previous studies have shown that functional recovery often occurs earlier than the time it would take to achieve functional integration of the transplanted NSCs, so other NSC‐mediated repair mechanisms such as trophic factor support may play a larger role in functional recovery than cell replacement (Oki et al., 2012). In addition, most preclinical rodent models show a rapid and high degree of spontaneous functional recovery making it challenging to identify functional improvements that occur 2 and 3 months post‐transplantation and would correlate with NSC integration. Identifying and separating the recovery effects of NSC trophic factor signaling from cell replacement are a unique and difficult challenge. However, it is generally hypothesized that functional integration of the grafted NSCs could lead to further neurological improvements albeit several months after transplantation (Tornero et al., 2013).

It is critically important that transplanted NSCs differentiate into region‐specific cell types and integrate appropriately. Aberrant incorporation of NSC derivatives could lead to abnormal connections causing seizure activity, pain, and other undesirable outcomes. However, it remains unclear whether the differentiation fate of grafted NSCs is driven by intrinsic mechanisms, environmental cues of the host tissue, or is spontaneous. To assess this question, Oki et al. (2012) transplanted NSCs into the cortex and striatum of stroked rodent brains. After 4 months, differentiated cells expressed the specific striatal projection neuron marker DARPP‐32 in both the cortex and striatum, indicating that spontaneous neuronal differentiation of grafted NSCs was not region‐specific. However, Snyder, Yoon, Flax, and Macklis (1997) demonstrated that selective layer II/III photolysis in the mouse neocortex prompted transplanted NSCs to differentiate into pyramidal neurons in a cortical layer‐appropriate manner while establishing appropriate synaptic contacts, indicating that external cues may have facilitated region‐specific cortical differentiation. It is possible that effective neuronal replacement in various brain regions will require directed in vitro differentiation of NSCs to site‐specific precursors prior to transplantation (Oki et al., 2012). Indeed, Tornero et al. (2013) found that cortically fated NSCs more readily differentiate to a cortical phenotype with pyramidal morphology than nonfated NSCs. Building upon this strategy, it may 1 day be possible to generate region‐specific cellular composites with the appropriate combination (e.g., specific neuron subtypes, astrocytes and oligodendrocytes) and transplant this cellular milieu with exquisite regionalized specificity. However, this strategy can become quite complex. For example, the cerebral cortex is composed of six unique layers that are comprised of different numbers and ratios of specialized neural cell types and changes depending upon brain region. In addition, this approach is also plagued by other complex variables such as brain vasculature remodeling involving multiple cell types (e.g., endothelial cells, pericytes, smooth muscle cells) as well as the need to form neurovascular units, and a lymphatic system (Benarroch, 2012; Louveau et al., 2015). Through the lens of NSC transplant studies in a number of neural injury and disease models, it has become clear that there is a need to better understand the mechanisms driving differentiation of grafted NSCs before cells can be safely and reliably used as a stroke therapy.

## 4 NEURAL STEM CELL‐CONDITIONED MEDIA

Recent studies have demonstrated that NSC culture‐conditioned media and purified media products inhibit apoptosis, reduce lesion size, and promote functional recovery in stroked preclinical models (Akerblom, Sachdeva, & Jakobsson, 2012; Delaloy et al., 2010; Madelaine et al., 2017; Stappert, Roese‐Koerner, & Brustle, 2015; Sutaria, Badawi, Phelps, & Schmittgen, 2017; Webb, Kaiser, Jurgielewicz et al., 2018; Webb, Kaiser, Scoville et al., 2018; Yang et al., 2018; Zhang et al., 2015). In a recent rat study utilizing NSC culture‐conditioned media directly, they showed a neuroprotective effect on tissue and functional improvement in a 21‐point behavioral test. These effects were attributed to NSCs releasing neurotrophic factors as well as micro‐ and nano‐sized extracellular vesicles (EVs) into the culture media. EVs derived from various cell sources have been shown to carry protein, DNA, and RNA cargoes that have therapeutic properties (Sutaria et al., 2017; Webb, Kaiser, Jurgielewicz et al., 2018; Zhang et al., 2015). Indeed, recent studies by Webb et al. demonstrated that EVs derived from NSCs mitigated the systemic immune response, reduced infarct volume, inhibited hemorrhagic transformation, improved white matter integrity, and promoted functional recovery in two divergent preclinical stroke models‐ mouse thromboembolic and pig permanent middle cerebral artery occlusion stroke model (Webb, Kaiser, Jurgielewicz et al., 2018; Webb, Kaiser, Scoville et al., 2018). Although the exact molecular mechanism of NSC EV‐induced recovery is unknown, flow cytometry analysis illustrated that NSC EVs harbor CD29 and CD41b, which may have played a role in promoting BBB integrity and partially explain the therapeutic effect (Webb, Kaiser, Jurgielewicz et al., 2018).

microRNAs, which are small RNAs that regulate gene expression at the post‐transcriptional level, can be found in NSC culture media and have been studied to better understand how they regulate NSC function and stroke outcome (Akerblom et al., 2012). For example, miR‐9 is expressed in neural progenitors and regulates neurogenesis, NSC migration, and angiogenesis through modulation of VEGF signaling (Delaloy et al., 2010; Madelaine et al., 2017; Stappert et al., 2015). The let‐7 family of microRNAs, which are also expressed by NSCs, have been shown to protect against neuroinflammation by regulating the expression of caspase 3, inducible nitric oxide synthase (iNOS), TNF‐α, and IL‐12, which improve stroke‐induced neurological deficits in mice (Akerblom et al., 2012; Banerjee et al., 2013; Ni et al., 2015). Furthermore, miR‐210 expression is upregulated in NSCs exposed to a hypoxic environment, and overexpression of this microRNA led to enhanced neurogenesis and angiogenesis in mice (Wang et al., 2013). Together, this indicates that NSC‐conditioned media is enriched with bioactive, restorative factors that are packaged into EVs or in their free form. These released factors leverage NSC therapies to a point in which whole cells may no longer be required in the future.

## 5 TISSUE ENGINEERING APPROACHES

While many reports outline the robust therapeutic effects of NSCs transplanted alone, recent evidence suggests that engineering approaches with biomaterials limits stroke‐induced tissue architecture disruptions and enhances NSC therapeutic function and engraftment (Bible, Qutachi et al., 2012; Jin, Mao et al., 2010; Lam, Lowry, Carmichael, & Segura, 2014; Lee, Yun, Park, & Jang, 2016; Yu et al., 2010). When cotransplanted with NSCs, the main role of biomaterials is often to cultivate an adequate structural microenvironment to foster the survival, cross talk, and integration of transplanted cells into host tissue. Furthermore, biomaterials can be enriched with neurotrophic growth factors to augment transplantation success (Bible, Qutachi et al., 2012). To this end, naturally occurring and synthetic biomaterials have been developed and co‐administered with NSCs in preclinical stroke models. A number of hydrogel materials seeded with NSCs have been designed and evaluated in preclinical stroke models including hyaluronic acid, collagen, Matrigel, and other xenogenic sources (Bible, Dell’Acqua et al., 2012; Jin, Mao et al., 2010; Jin et al., 2011; Lam et al., 2014; Lee et al., 2016; Moshayedi et al., 2016; Yu et al., 2010). These studies show that transplanted cells survive quite well in hydrogels due to excellent nutrient and oxygen permeability, and co‐administration of NSCs with hydrogel reduces infarct size, increases host neurogenesis, and promotes functional recovery. However, cell migration in hydrogels is often poor due to weak mechanical structure, and neurons do not extend their neurites through these three‐dimensional matrices efficiently (Skop, Calderon, Cho, Gandhi, & Levison, 2014).

Opposed to hydrogels which are soft and become gelatinous upon brain injection, synthetic microparticles have a rigid structure on which neuronal growth cones can be sustained more efficiently (Park, Teng, & Snyder, 2002; Skop et al., 2014). Bible et al. (2009) transplanted a scaffold consisting of plasma polymerized allylamine (ppAAm)‐treated poly(D,L‐lactic acid‐co‐glycolic acid; PLGA) particles along with NSCs into the lesion cavity of stroked rats. Utilizing MRI to monitor integration of the NSC‐scaffold matrices, they demonstrate primitive de novo tissue formation within 7 days post‐transplantation. Subsequent histological analysis showed that the graft was fibrous in appearance along the periphery and consisted of neurons and astrocytes. However, the newly formed tissue was completely void of vasculature that could sustain long‐term viability. In a follow‐up study, the group demonstrated that encapsulating the PLGA microparticles with VEGF promotes endogenous endothelial cell migration to the graft site which contributes to neovascularization (Bible, Qutachi et al., 2012). These findings were corroborated by Yamashita et al. who reported an increased number of endothelial cells and astrocytes after the administration of VEGF‐enriched scaffold into the stroke cavity of their animal model, which led to increased tissue volume in the graft (Yamashita, Deguchi, Nagotani, & Abe, 2011). Future studies evaluating the benefit of enriching biomaterials with other potent neurotrophic factors, such as BDNF, GDNF, and NGF, would be useful to elucidate what signaling cascades will maximize NSC transplantation success.

## 6 CLINICAL CONSIDERATIONS FOR TRANSLATION

There are a number of challenges to address before NSC therapies can be widely adopted for clinical stroke treatment—many of which have yet to be assessed. The stroke brain environment is truly unique with respect to immune system challenges relative to studying cell replacement therapies in other organs. The brain is typically immunoprivileged, yet in the stroke environment the BBB is compromised and undergoes dynamic changes in permeability allowing for infiltration of systemic immune cells. This has raised questions pertaining to the immune system response to transplantation of allogeneic NSC lines in stroke and the potential need for autologous iPSC‐NSCs to overcome this response (Manley et al., 2015). However, the amount of time needed to generate and safety test iPSC‐NSCs with current technologies is likely beyond the treatment window of therapeutic efficacy if one expects large‐scale cell replacement. It is unclear the extent that additional factors such as stroke type, localization, and severity effect the success of NSC transplantation. Furthermore, comorbidities commonly encountered with stroke patients (e.g., diabetes and hypertension) as well as age effects and sex have not been or only limitedly assessed for their effects on NSC therapies in stroke. Indeed, only a handful of preclinical stroke studies are conducted in aged animals despite documented evidence of varying effectiveness of stroke therapeutics between young and aged animals (Popa‐Wagner, Filfan, Uzoni, Pourgolafshan, & Buga, 2015; Sandu et al., 2017; Webb, Kaiser, Scoville et al., 2018). Likewise, while animal models of diabetes, hypertension, and vascular dementia exist, they are rarely used to test NSC therapeutics in stroke in the preclinical setting. Many of these common comorbidities are associated with low‐grade neuroinflammation which could directly impact the success of NSC therapy (Sandu, Buga, Uzoni, Petcu, & Popa‐Wagner, 2015). In order to overcome translational barriers between preclinical success and clinical trials going forward, more emphasis should be placed on optimizing NSC‐based therapies in “real‐world” animal models. Nonetheless, three clinical factors that have been assessed, albeit not typically in a direct fashion, are route of administration, dose, and treatment window.

### 6.1 Route of administration

A variety of NSC administration routes have been described for stroke including intraparenchymal (IP) and intravenous (IV) transplantation. In preclinical animal models of stroke, IP injections are the most commonly reported and are performed through a transcranial approach (Table 1). The popularity of IP transplantation is likely due to the numerous advantages of direct transplantation including site specificity, eliminating the need for cells to transverse the BBB, guaranteed delivery of large cell numbers to the injury site, and limiting the potential for off‐target effects (e.g., clogging microvasculature in nontarget organs leading to ischemic events; Lau et al., 2015). The most significant hurdle to the clinical use of IP transplantation is the highly invasive nature of the required craniectomy, which could lead to additional complications in stroke patients that are already severely compromised. However, two recent clinical trials testing the safety and efficacy of IP transplantation of NSCs (Kalladka et al., 2016) and adult stem cells (ASCs; Steinberg et al., 2016) demonstrated that transcranial surgery resulted in minimal adverse events and was generally safe and well tolerated, indicating that IP delivery of NSCs is deserving of further clinical study.

IV administration of NSCs is another popular route since it is generally the least invasive and is less technically challenging (Lau et al., 2015). Indeed, IV administration could potentially allow NSCs to be injected during the acute stage of stroke when patients may be too unstable to undergo transcranial IP NSC treatment, or enable NSCs to be administered by healthcare professionals that are not capable of performing a craniotomy. Thus, IV NSC treatment could be employed by paramedics during the ambulance ride to the hospital or utilized in rural areas where neurosurgery expertise is limited. However, IV transplantation results in less cell engraftment in the brain due to the propensity of cells to accumulate systemically in nontarget organs such as the lungs and liver (Fischer et al., 2009; Lappalainen et al., 2008). Previous studies have shown that IV NSC delivery results in neuroprotection and improved neurological performance without evidence of cell engraftment in the stroked brain, indicating that anti‐inflammatory and regenerative trophic factor release is the main mechanism behind this treatment strategy (Watanabe et al., 2016). Taken together, safety considerations along with intended NSC therapeutic action should be considered when developing effective, translatable NSC therapies for stroke.

### 6.2 Treatment window and dosing

A wide range of NSC treatment windows have been assessed in rodent models, ranging from immediately after reperfusion to 4 weeks post‐stroke (Table 1). In these preclinical studies, the treatment window is often dictated by the predicted mode of therapeutic action. If the main objective is to maximize the neuroprotective and immunomodulatory roles of transplanted NSCs, then transplantation often occurred within the acute stage of stroke before infarction is complete and tissue can be rescued (Kokaia, Andsberg, Martinez‐Serrano, & Lindvall, 1998). However, a recent clinical trial of ASC transplantation in chronic stroke patients with stable neurological function scores demonstrated significant improvement in mean scale scores likely through ASC‐mediated neuroprotective and regenerative mechanisms, indicating that tissue remodeling may still be active several months post‐stroke in which stem cell therapy can augment tissue recovery through trophic factor signaling (Steinberg et al., 2016). Conversely, for cellular replacement strategies, a transplantation time point in the less cytotoxic subacute to chronic stages may be more advantageous to enhance long‐term survival of the grafted cells. Darsalia et al. reported that NSC transplantation 48 hr post‐stroke improved cell survival compared to 6 weeks post‐stroke; furthermore, the delayed transplantation did not augment NSC migration, proliferation, or neuronal differentiation (Darsalia et al., 2011). Another technical consideration that warrants further study for clinical translation is the optimal NSC dose (i.e., number of cells and number of transplantations). In rodent studies, cell dosing ranges from 1.0 × 10^5^ to 4.5 × 10^5^ cells in one to three transplantation sites for IP and a single dose of 3.0 × 10^6^ to 4.0 × 10^6^ cells for IV with no evidence of outcome discrepancies between transplantation protocols. Indeed, Darsalia et al. (2011) demonstrated that transplanting a greater number of NSCs does not result in a higher number of grafted cells or increased neuronal differentiation. Taken together, this suggests that an early treatment time window, before the inflammatory response is established, may be a more important factor determining engraftment success compared to dosing.

## 7 CONCLUSION

NSCs provide the unique opportunity to mitigate stroke pathology through multimodal therapeutic action. A number of preclinical studies in rodent stroke models have demonstrated promising evidence that NSCs are able to act as a neuroprotectant by limiting secondary injury through anti‐inflammatory mechanisms, promoting endogenous neurogenesis and synaptic remodeling, and even act as a cell replacement thereby promoting tissue and functional recovery. These preclinical findings have led to human clinical trials assessing NSC safety and efficacy in stroke patients with promising results. However, additional studies designed to better understand important factors determining NSC engraftment success such as cotransplantation of multiple cell types, treatment time window, dosing number, and the effect of age and comorbidities will ultimately augment therapeutic efficacy and hopefully improve stroke prognosis and future treatment paradigms.

## CONFLICT OF INTEREST

The authors declare no conflicts of interest.
